# Bacterial succession and metabolite changes during flax (*Linum usitatissimum* L.) retting with *Bacillus cereus* HDYM-02

**DOI:** 10.1038/srep31812

**Published:** 2016-09-02

**Authors:** Dan Zhao, Pengfei Liu, Chao Pan, Renpeng Du, Wenxiang Ping, Jingping Ge

**Affiliations:** 1 Laboratory of Microbiology, College of Life Science, Heilongjiang University, Harbin, China; 2 Engineering Research Center of Agricultural Microbiology Technology, Ministry of Education, Heilongjiang University, Harbin, China.

## Abstract

High-throughput sequencing and GC-MS (gas chromatography-mass spectrometry) were jointly used to reveal the bacterial succession and metabolite changes during flax (*Linum usitatissimum* L.) retting. The inoculation of *Bacillus cereus* HDYM-02 decreased bacterial richness and diversity. This inoculum led to the replacement of Enterobacteriaceae by Bacillaceae. The level of aerobic Pseudomonadaceae (mainly *Azotobacter*) and anaerobic Clostridiaceae_1 gradually increased and decreased, respectively. Following the addition of *B. cereus* HDYM-02, the dominant groups were all degumming enzyme producers or have been proven to be involved in microbial retting throughout the entire retting period. These results could be verified by the metabolite changes, either degumming enzymes or their catalytic products galacturonic acid and reducing sugars. The GC-MS data showed a clear separation between flax retting with and without *B. cereus* HDYM-02, particularly within the first 72 h. These findings reveal the important bacterial groups that are involved in fiber retting and will facilitate improvements in the retting process.

Flax (*Linum usitatissimum* L.) is a fiber-bearing plant and has been widely grown and exploited for thousands of years. Flax fiber is one of the oldest natural bast fibers and has been used in the textile, material and medical fields because of its desirable properties^1^,^2^,^3^. Products made of flax fiber, as well as fibers from other plants such as hemp, kenaf, ramie and jute, can be substituted for fiberglass/epoxy materials and are thus environmentally friendly and helpful to relieve the petroleum crisis. The separation of bast fibers from plant straws can be achieved using to mechanical or chemical processes, which usually result in poor-quality fibers^4^,^5^. Retting is widely applied to extract bast fibers from either straws or decorticated fibers using enzymes produced by microbes to degrade the gummy substances linking bast fibers together such as pectin, lignin, hemicellulose and wax^6^. Retting with commercial enzymes is likely cost-prohibitive, although high-quality fibers are produced^7^. Therefore, microbial retting offers the best chance for the large-scale production of high-quality fibers.

Several degumming enzyme producers, particularly pectinolytic bacterial strains, including *Bacillus*, *Clostridium* and *Pseudomonas*, have been isolated and re-inoculated into retting ecosystem, resulting in shorter retting time and better fiber qualities^8^,^9^,^10^,^11^,^12^. Recently, several researchers have identified the bacterial community in the retting ecosystem using modern molecular techniques, such as 16S gene library^13^ and denatured gradient gel electrophoresis (DGGE)^12^,^14^,^15^, and have shown that the common dominant groups present during retting are Clostridiaceae, Pseudomonadaceae and Bacillaceae. Visi *et al.* used next-generation semiconductor sequencing to conclude that the community structure during kenaf retting the was first driven by the switch to anaerobic conditions and subsequently by possible competition for nitrogen^16^. Retting is a substantially complicated ecosystem that involves interaction between biotic and abiotic factors, i.e., microbes and metabolites. Investigations of the relationship between bacterial succession and metabolite changes are the best way to understand and improve the retting process. However, little is known about the specific microbes involved in degumming, the process for degrading gummy substances and the dynamic amendment of inoculated strains to retting solution.

In this research, *B. cereus* HDYM-02 was used as an inoculum to estimate its effects on the transition of both the bacterial community and metabolites. *B. cereus* HDYM-02, isolated from flax retting solution, was a degumming enzyme producer^17^, which conferred its biocompatibility in practical applications of microbial retting of flax straws^18^,^19^,^20^. For the first time, high-throughput sequencing and gas chromatography-mass spectrometry (GC-MS) were jointly used to reveal the relationship between bacterial succession and the metabolite changes during flax retting. The results derived from this study discovered dominant and unreported bacterial groups that were involved in retting and offered a profile of the metabolite transitions. These findings suggested the possibilities for facilitating improvements to the retting process, with shorter times and better fiber qualities.

## Materials and Methods

### Microorganism and culture

Bacterial strain *B. cereus* HDYM-02 was isolated from a flax retting solution^21^ and stored in 30% glycerol at −80 °C. Fresh media was inoculated with the −80 °C freezer stocks, and the cultures were grown overnight in 250 ml of Luria-Bertani medium in 500-ml Erlenmeyer flasks at 37 °C with shaking. Cells were collected by centrifugation, suspended and adjusted to 10^8^ cells/ml in tap water as the microbial inoculum for flax retting.

### Flax retting

Fibrous flax seeds were purchased from Heilongjiang Academy of Agriculture Sciences, China, and had been homogenized by the seller such that the seeds were even and similar in shape and size. The seeds were planted in black soil of an experimental field at East University of Heilongjiang, China, N 45°66 and E 126°36, where the active accumulated temperature was in the range of 2700 to 3400 °C. Straws were harvested 105 days post-planting during May to mid-August and then dried in the field. The middle part of flax straws was chosen for retting and cut to a length of approximately 600 mm, with a diameter of approximately 1.3 mm to achieve uniformity across all experiments and ensure that the straws would fit within the retting tank. The retting experiments were performed in 30 L aluminum tanks filled with flax straws and tap water at a ratio of 1:15 at 35 °C for 120 h. The microbial retting tank was denoted as BA and was inoculated with 5 × 10^8^ *B. cereus* HDYM-02 cells per gram of flax straw. The control tank without the microbial inoculum underwent natural retting and was denoted as CK. In this study, each retting experiment was performed in six independent replicates.

### Protein and enzyme assays

The total extracellular protein content was determined based on the Lowry procedure using bovine serum albumin (BSA) as the standard^22^. The pectinase activity was determined using the method described by Sampriya *et al.*^23^. Ten microliters of properly diluted crude enzyme were added to 490 μl of a 0.1% pectin solution and incubated at 65 °C for 5 min. Then, 1.5 ml of dinitrosalicylic acid was added to the reaction mixture, which was heated up in boiling water for 15 min. The absorbance was measured at 540 nm. One unit of enzyme activity was defined as the amount of enzyme that released 1 μg of galacturonic acid per minute under certain conditions. The mannanase activity was determined using the method reported by Akino *et al.* with slight modifications^24^. The reaction mixture, which consisted of 0.1 ml of properly diluted crude enzyme and 0.9 ml of reducing sugar-free konjaku flour (0.5%, w/v), was incubated at 55 °C for 30 min. Then, 3 ml of dinitrosalicylic acid was added to the reaction mixture, which was heated up in boiling water for 5 min. The absorbance was measured at 550 nm. One unit of activity was defined as the amount of enzyme that produced 1 μmol of mannose per minute.

### DNA extraction, PCR amplification and high-throughput sequencing

Three out of six randomly selected retting solution samples were collected at 24, 48, 72, 96 and 120 h, respectively. The community DNA was extracted with the TIANamp Bacteria DNA Kit (DP302) (TIANGEN, China) according to the manufacture’s protocols. The DNA extracts were stored at −20 °C prior to PCR amplification. The V4 and V5 hypervariable regions of the 16S rRNAs (*Escherichia coli* positions 515–907) were selected for PCR. The primers were 515F (5′-GTGCCAGCMGCCGCGG-3′) and 907R (5′-CCGTCAATTCMTTTRAGTTT-3′), where the barcode is an eight-base sequence unique to each sample^25^. The PCR reactions were performed in triplicate 20-μL mixtures containing 4 μL of 5× FastPfu Buffer, 2 μL of 2.5 mM dNTPs, 0.8 μL of each primer (5 μM), 0.4 μL of FastPfu polymerase, and 10 ng of template DNA. The PCR procedure consisted of an initial 2 min denaturation at 95 °C; 25 cycles of denaturing at 94 °C for 30 s, annealing at 55 °C for 30 s, and extension at 72 °C for 30 s; and a final extension at 72 °C for 5 min.

The amplicons were extracted from 2% agarose gels and purified using the AxyPrep DNA Gel Extraction Kit (Axygen Biosciences, Union City, CA, U.S.) according to the manufacturer’s instructions and quantified using QuantiFluor™ -ST (Promega, U.S.). The purified amplicons were pooled in equimolar amounts and paired-end sequenced (2 × 250) on an Illumina MiSeq platform according to standard protocols. The raw reads were deposited into the NCBI Sequence Read Archive (SRA) database (Accession Number: SRP068887).

### Sequence analysis and phylogenetic classification

The raw fastq files were demultiplexed and quality-filtered using QIIME (version 1.17)^26^. The operational Units (OTUs) were clustered with a 97% similarity cutoff using UPARSE (version 7.1 http://drive5.com/uparse/)^27^ and chimeric sequences were identified and removed using UCHIME^28^. The phylogenetic affiliation of each 16S rRNA gene sequence was analyzed by RDP Classifier (http://rdp.cme.msu.edu/)^29^ against the silva (SSU115)16S rRNA database using a confidence threshold of 70%^30^. The clusters were constructed at a 3% dissimilarity cutoff and served as OTUs for generating predictive rarefaction models and for determining the ACE (abundance-based coverage estimators) and the Chao 1 richness and Shannon-Weaver diversity indices. The interrelationships between the bacterial communities from both the BA and CK samples were visualized using PCA (principle component analysis).

### Metabolite analysis

All supernatants of the retting solution samples were lyophilized and dissolved in 200 μl of pyridinamine (150 mg/ml) and 200 μl of N-methyl-N-(trimethylsilyl)trifluoroacetamide (containing 1% trimethylchlorosilane), and incubated at 70 °C for 1 h. Then, the samples were mixed with 300 μl of dichloromethane and filtered with 0.22-μm filtration membranes. The resulting solutions were transferred into tubes and their GC-MS spectra were obtained using a 7890A/5975C GC-MS spectrometer (Agilent, USA). Individual metabolites from the GC-MS spectra were identified and quantified using the Agilent OpenLAB CDS Chemstation. The metabolites were identified by searching the NIST11.5 database and converted to AIA form. The AIA data were processed, including data extraction, peak matching, Rt adjustment, visualization and normalization, using R 3.1.3 (Smooth Sidewalk, released on 2015-03-09) for the multivariate statistical analysis. The PCA model was established and verified with SIMCA-P 11.5. The reducing sugar content was determined by the DNS method^31^. Galacturonic acid was assayed using the method of Dietz and Rouse^32^.

### Statistical analysis

Three samples out of the six replicates were used for high-throughput sequencing. The data represent the mean values of three independent replicates ±SD (standard deviation) at each time point. All six samples were used for GC-MS and the enzyme and protein assays. The data represent the mean values of the six independent replicates ±SD at each time point. Differences between groups were compared using ANOVA and Tukey’s test. Differences were considered significant when the p value was less than 0.05. The data were statistically analyzed using JMP 9.0.2 (SAS Institute Inc., USA).

## Results

### Overall bacterial phylogeny and community diversity

In total, we obtained 614,136 quality sequences from six retting solution samples (three BA and three CK) at five time points (24, 48, 72, 96 and 120 h), and an average of 16,558-28,409 sequences were obtained per sample (mean = 20,471). The read lengths ranged from 301 to 400 with an average of 396. The sequence information and calculated bacterial diversity index were listed in Table 1. The mean values of ACE, Chao1 and Shannon-Weaver values in the BA samples were significantly lower than those in the CK samples, which indicated a decrease in both bacterial richness and diversity with the of addition of *B. cereus* HDYM-02. The results coincided with the slight decrease in the average sums of reads and OTUs in the BA samples (101, 588 reads, 346 OTUs) compared with the CK samples (103, 124 reads, 349 OTUs).

### Bacterial community structure

**Figure 1:**
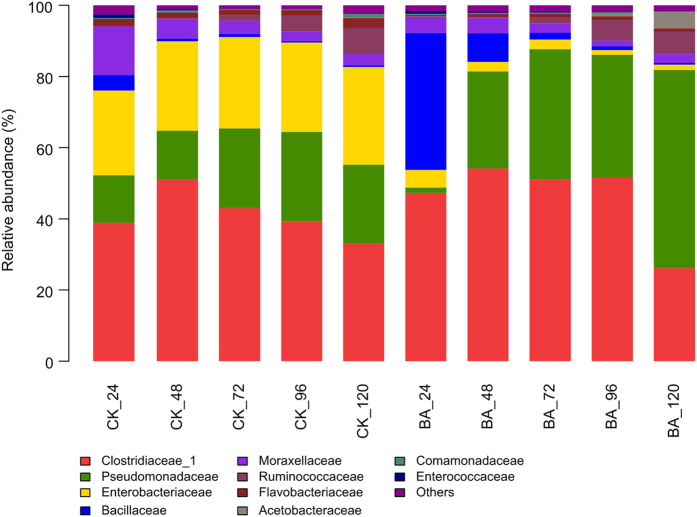
Bacterial community structures at family level. The abundance is presented in terms of a percentage of bacterial sequences in retting solution samples.

**Figure 2:**
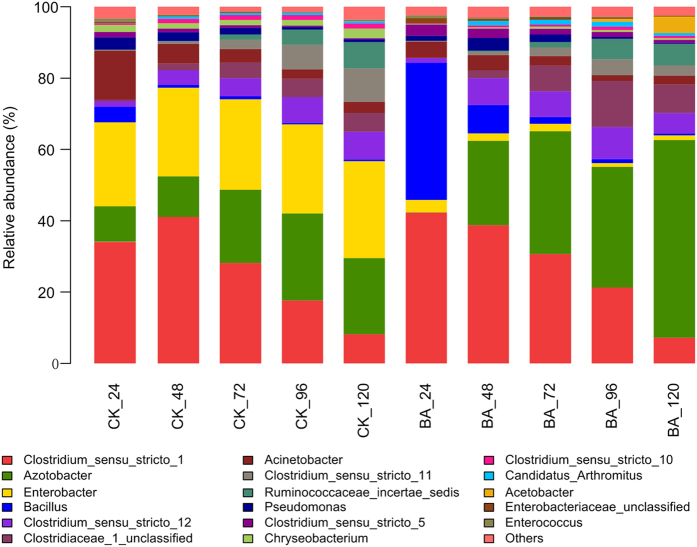
Bacterial community structures at genus level. The abundance is presented in terms of a percentage of bacterial sequences in retting solution samples.

The five phyla identified in both the BA and CK samples were Actinobacteria, Cyanobacteria, Bacteroidetes, Firmicutes and Proteobacteria. The latter three phyla were predominant and comprised the following eight classes: Flavobacteriia, Sphingobacteriia, Bacilli, Clostridia, Negativicutes, Alphaproteobacteria, Betaproteobacteria and Gammaproteobacteria. These eight classes were subdivided into fourteen orders: Flavobacteriales, Sphingobacteriales, Bacillales, Lactobacillales, Clostridiales, Selenomonadales, Caulobacterales, Rhizobiales, Rhodospirillales, Sphingomonadales, Burkholderiales, Enterobacteriales, Pseudomonadales and Xanthomonadales. The bacterial communities at the family and genus levels were shown in Figs 1 and 2, respectively. The common, predominant bacteria present in all of the retting solution samples were represented by the families Clostridiaceae and Pseudomonadaceae. Enterobacteriaceae comprised a relatively large proportion of the community throughout naturally retting process, i.e., CK samples, which was replaced by Bacillacea within the BA_24 sample due to the addition of *B. cereus* HDYM-02. The bacterial compositions were similar between the BA and CK samples. Nevertheless, the abundance of several families was distinguished by the addition of *B. cereus* HDYM-02. The most remarkable difference was the increased Bacillaceae (average abundance in BA: 10.0% and CK 1.4%) and decreased Enterobacteriaceae (average abundance in BA: 2.6% and CK 25.4%) abundances. Moreover, the average abundance of Pseudomonadaceae and Clostridiaceae was enhanced in the BA samples (31.1% and 46.1%, respectively) as compared to the CK samples (19.4% and 41.0%, respectively).

**Figure 3:**
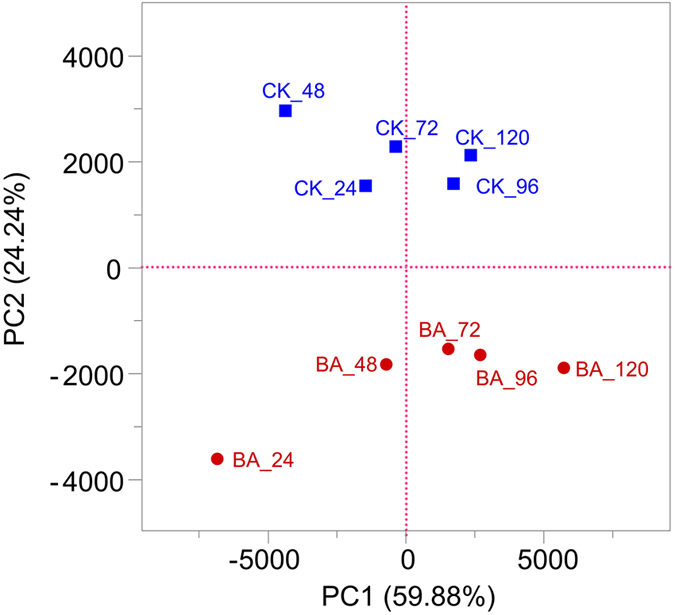
PCA analysis of retting solution samples based on the composition of bacterial communities.

As shown in Fig. 3, PCA analysis revealed a significant separation of the bacterial communities between the BA and CK samples. The cumulative percentage variance of the species explained by PC1 and PC2 were 59.88% and 24.24%, respectively. Following the addition of *B. cereus* HDYM-02, the BA_24 h sample obviously separated from the other nine samples. Enterobacteriaceae, Clostridiaceae and Pseudomonadaceae represented the largest three contributions to the PCA (Table 2). These three families were also predominant and notably fluctuated in the retting solution samples (Fig. 2).

### Changes in metabolites

**Figure 4:**
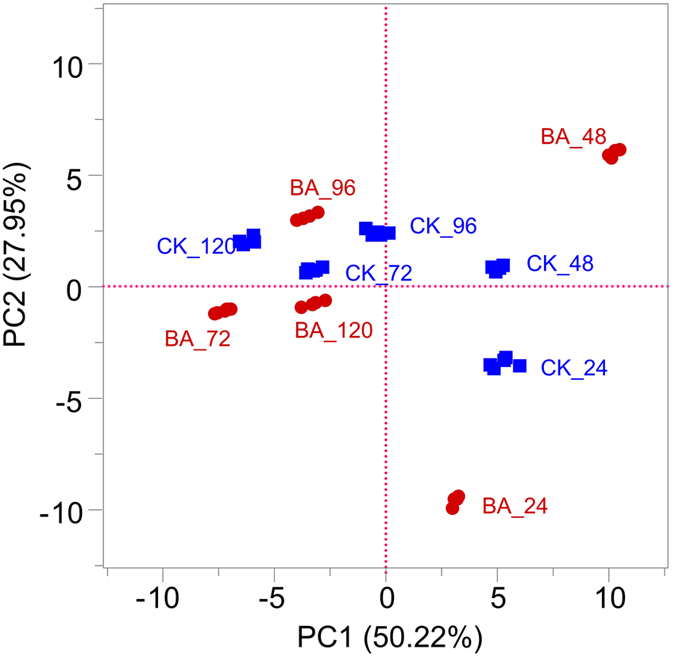
PCA based on GC-MS spectra of metabolites obtained from the retting solution samples.

**Figure 5:**
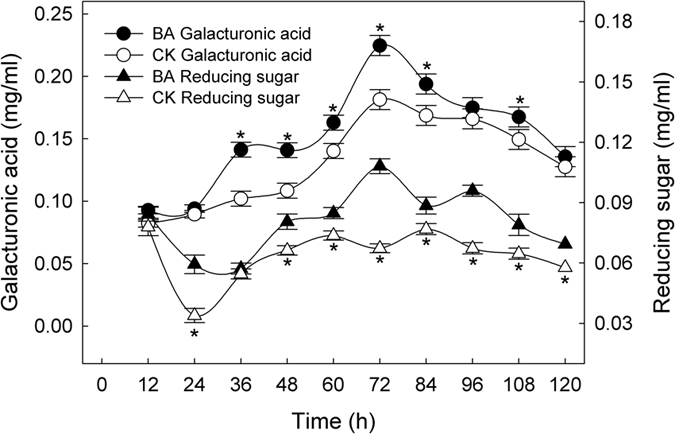
Changes in galacturonic acid and reducing sugar of retting solution samples. *Indicate significant differences between BA and CK samples at each time point.

A total of 460 organic metabolites were identified based on the GC-MS spectra from all of the retting solution samples, including hydrocarbons and their derivatives, acids, esters, sterols, aldehydes, and aromatics. A PCA was performed using all metabolites identified in GC-MS spectra to examine the effects of *B. cereus* HDYM-02 on the variations of metabolites during the entire retting period, a (Fig. 4). Two principal components explained 78.17% of the total variance. The PCA plots showed differences between the BA and CK samples. Generally, the spots shifts indicated that continuous metabolic changes occurred during the retting process. Spots representing the BA samples were more scattered than those representing CK samples, suggesting faster succession and a different composition of metabolites. Retting processed very rapidly between 24 h to 72 h, particularly for the BA samples, which was in accordance with the galacturonic acid profile shown in Fig. 5. Thereafter, the spots moved slowly until 120 h, indicating that the metabolites in the retting solution samples changed slowly during this period. Nineteen out of 460 metabolites were considered as significantly changed during retting the period based on the VIP (variable importance in the projection) and P values (Supplemental Table 1).

Galacturonic acid and reducing sugars, residues of gummy substances present in flax straw, were selected as indicators of the extent of degumming during retting process (Fig. 5). Generally, the dynamic changes in these residues showed two coincident features: fluctuations in the wave pattern, i.e., the rise-fall-rise-fall cycle, and higher concentrations in the BA samples than in the CK samples. In the BA and CK samples, the peak values of galacturonic acid were 0.22 mg/ml and 0.18 mg/ml at 72 h, respectively, whereas the peak values of the reducing sugars were 0.11 mg/ml at 72 h and 0.08 mg/ml at 84 h, respectively.

### Changes in degumming enzymes and proteins

**Figure 6:**
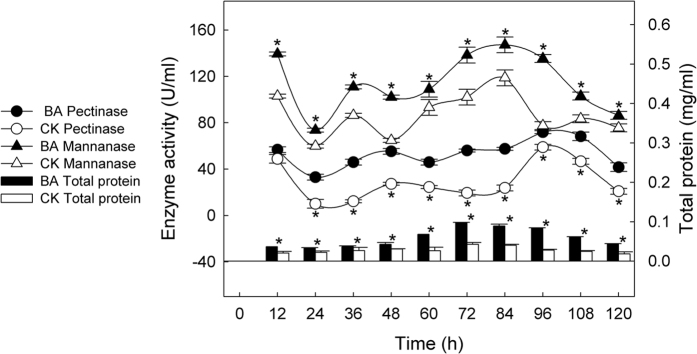
Changes in degumming enzymes and extracellular total protein of retting solution samples. *Indicate significant differences between BA and CK samples at each time point.

The dynamic changes in the degumming enzymes, including pectinase and mannanase, and proteins were illustrated in Fig. 6. A similar wave pattern as that of galacturonic acid and the reducing sugars shown in Fig. 5 was observed. The BA samples consistently displayed higher enzyme activity than the CK samples. The peak values for pectinase were 71.86 U/ml in the BA_96 h sample and 58.92 U/ml in the CK_96 h sample, whereas the peak values for mannanase were 147.21 U/ml in the BA_84 h sample and 118.75 U/ml in the CK_84 h sample. Degumming enzymes were the main extracellular proteins in the retting solutions. Consequently, the protein concentration was generally higher in the BA samples than in the CK samples. The highest values appeared at 72 h and were 0.10 mg/ml and 0.04 mg/ml, respectively.

## Discussion

### Bacterial succession

A small number of studies have used modern molecular techniques based on the 16S rRNA to explore the bacterial communities in retting solutions of bast fiber plants, such as clone library in jute^13^ and DGGE in jute^15^ and flax^12^,^14^. Recently, one high-throughput sequencing platform, semiconductor sequencing, has been applied in investigations of the bacterial retting community of kenaf under different retting conditions at the order level^16^. In this study, high-throughput sequencing was first used to reveal the bacterial succession from phylum to species during the entire retting period for flax straw. Two basic retting experiments were initially conducted. CK was performed to mimic and reveal a natural retting process, including the contributions of the inherent microbes present in flax straws and tap water. BA was performed to estimate the effects of the bacterial strain *B. cereus* HDYM-02 on bacterial succession, structure and transition.

As shown in Fig. 2, although the bacterial composition at the family level was very similar between the CK and BA samples, the abundances among the bacterial genera were clearly changed following the addition of *B. cereus* HDYM-02, as expected. Enterobacteriaceae represented a large proportion of the community in the CK samples throughout the entire retting period, suggesting that it was naturally associated with the flax straw. In the BA samples, Bacillaceae almost totally replaced Enterobacteriaceae within 24 h, which almost completely disappeared throughout the whole retting period. The competition for oxygen and nutrients resulted in the replacement of Enterobacteriaceae by Bacillaceae, in accordance with the replacement of the inherent Entrerobacteriales by Bacillales after inoculating *Bacillus* and *Paenibacillus* strains during kenaf retting^16^. In this study, the competition among aerobes following inoculation with *B. cereus* HDYM-02 also led to the decrease of *Chryseobacterium*, Flavobacteriaceae, and *Acinetobacter*, Moraxellaceae (Fig. 3), two families that were also found in flax retting solutions using DGGE^12^.

The other dominant family in this research was Pseudomonadaceae (Fig. 1), which was subdivided into *Azotobacter* and *Pseudomonas* (Fig. 2). *Pseudomonas* is the dominant genus involved in the microbial retting of flax^12^,^14^ and kenaf^16^. Nevertheless, in this study, the average abundance of *Pseudomonas* was less than 2.0% across all of the retting samples. *Azotobacter* was unexpectedly found to be the major component of Pseudomonadaceae. Additionally, the average abundance of *Azotobacter* was significantly enhanced by the addition of *B. cereus* HDYM-02, i.e., 17.5% in the CK and 28.0% in the BA samples. *Azotobacter* widely exists in soil as a nitrogen fixer under aerobic conditions, but has never been identified in flax retting solutions. Surprisingly, in this study, *Azotobacter* rather than *Pseudomonas*, gradually became the dominant group, particularly in the BA samples (55.4%, BA 120 h). It maybe speculated that *Azotobacter* thrived to cope with the nutrient depletion by fixing nitrogen. Notably, *Azotobacter* has been reported as consortium component applied in microbial retting for coir fiber^33^, suggesting its retting capacity. An in-depth investigation of this group with respect to its succession and function during flax retting must generate further interest.

The family Clostridiaceae_1 was found to be another major microbial group in both the CK and BA samples during the entire retting period (Fig. 1), indicating their naturally association with the flax straw, similar to kenaf^16^. Short-length sequencing always loses accuracy as it moves lower in the taxonomic arrangement (i.e., more sequences start to be unclassified), which resulted in the assignment of several *Clostridium sensu stricto* rather than specific species (Fig. 2). As retting proceeded, several reports showed a later colonization of strictly anaerobic Clostridiaceae, such as *C. acetobutylicm* and *C. felsineum*, due to the environmental transition from aerobic to anaerobic conditions^11^. Visi *et al.* concluded that at the fourth day of kenaf retting, *Clostridium* was established and began fixing nitrogen as oxygen levels decreased and nutrient were depleted. Moreover, the heavy inoculation of *Bacillus* could accelerate the establishment of these bacteria^16^. Similar results were observed in this study, i.e., an apparent increase in Clostridiaceae abundance occurred from 24 h to 48 h in both the CK (38.9% to 51.0%) and BA (47.3% to 54.2%) samples. However, as retting proceeded, the levels of the anaerobic *Clostridium* gradually declined. Alternatively, the levels of the aerobic *Azotobacter* increased and acted as a nitrogen fixer, similar to *Clostridium*, indicating that the retting system functioned well. This discrepancy might result from the different experimental conditions. The retting experiments were performed in tanks without covers, unlike those performed in airtight containers^16^.

### Changes in metabolites

As shown in Fig. 4, the PCA score plots of all metabolites from the GC-MS-based metabolic analysis showed clear differences in retting extents between the samples retted with and without *B. cereus* HDYM-02. Generally, the points representing the BA samples were more scattered than those representing the CK samples, indicating that more apparent transitions in the metabolites resulted from the addition of *B. cereus* HDYM-02. The distances among the points representing BA samples were much more remote, which meant that flax retting with *B. cereus* HDYM-02 proceeded faster, particularly during the first 72 h. As retting proceeded from 72 to 120 h, the points gradually clustered, i.e., the differences in metabolites between the BA and CK samples were reduced.

Six categories of lignin and wax residues, including 4,6-dimethyl-dodecane, tetradecanoic acid, phthalic acid, n-hexadecanoic acid, 4-(2-propenyl)-phenol, and octadecanoic acid, originating from the surface and inside of the bast fiber^6^ showed the largest abundances at 72 h, which were significantly larger in the BA samples than in the CK samples (Supplemental Table 1). These available results revealed that the lignins and waxes inside the flax straws were degraded more thoroughly following the addition of *B. cereus* HDYM-02, suggesting a more effective retting within 72 h, particularly in the BA samples. In addition to fungi, bacteria are also important decomposers of lignins^34^ and waxes^35^. Several *Bacilli* have been proven to be lignin-degraders^36^,^37^, including *B. cereus*^38^. In this study, the other dominant retting species *Azotobacter* (Fig. 2), also has once been reported to be able to grow within lignin as carbon source^39^. Moreover, *Azotobacter* are capable of degrading many molecules with complicated structures, such as phenols^40^,^41^, carbohydrates^42^ and organic acids^43^. It could be speculated that *Azotobacter* played an important role in the retting process by degrading lignins and waxes. The data obtained from the GC-MS spectra should be further explored to identify some metabolites as biomarkers of certain phases of the retting period.

Pectin and hemicellulose are the main gummy substances present in the straws of fiber plants, which can be degraded by the degumming enzymes produced by microbes such as pectinolytic bacteria^10^,^44^, and mannanase- and xylanase-producing bacteria^45^,^46^. Afterwards, the presence of galacturonic acid and reducing sugars, which are residues of gummy substances, can indicate the degumming extent^47^,^48^,^49^. Both galacturonic acid and reducing sugars were maintained at higher concentrations in the BA samples than in the CK samples throughout the entire retting period (Fig. 5), which, consequently, were consistent with the degumming enzymes and total protein concentrations (Fig. 6).

This result suggested that there was a more thorough removal of gummy substances within flax straws in the BA samples, which could be explained by the bacterial succession (Figs 1 and 2). When retting proceeded to 24 h, the discrepancy was clear because Bacillaceae, mainly *Bacillus*, almost completely replaced Enterobacteriaceae in the BA samples. The *B. cereus* HDYM-02 inoculum together with many other *Bacilli*, have been proven to produce degumming enzymes, such as pectinase, pectate lyase, mannanase and xylanase^50^,^51^,^52^,^53^. In contrast, members of Enterobacteriaceae rarely produce degumming enzymes. As retting proceeded from 48 h to the end of the process, the most distinct feature in the BA samples compared with the CK samples was the increase in *Azotobacter*. To date, no studies have directly reported this genus as a degumming enzyme producer. However, evidence shows that *Azotobacter* can promote the pectinolytic quantities during the composting of agro-industrial refuse^54^. In addition, *Azotobacter* has been reported as consortium component applied to microbial retting for coir fiber^33^, suggesting its retting capacity. Given its abundant proportion and apparent importance during retting, *Azotobacter* warrants further investigation.

In addition, another notable group was Paenibacillaceae, whose members showed a slightly higher abundance in the BA samples, including *Paenibacillus*, *Brevibacillus* and *Saccharibacillus. Paenibacillus* has been a promising species in the field of microbial retting^16^ because numerous isolates have been found to produce varying types of pectinases^55^. Moreover, *Paenibacillus* can produce antibiotics, which may lead to the changes in the bacterial composition during the retting process^56^. Pectate lyase activity has been detected in *Brevibacillus* and *Saccharibacillus*, suggesting that these bacteria can degrade pectin^57^. Therefore, it could be speculated that the Paenibacillaceae present in the BA samples had a greater contribution to the degradation of gummy substances during the retting period to some extent.

In short, this study showed that flax retting with *B. cereus* HDYM-02 as an inoculum significantly changed bacterial succession and efficiently accelerated the retting process compared to natural retting. Following the addition of *B. cereus* HDYM-02, Bacillaceae almost totally replaced Enterobacteriaceae. Pseudomonadaceae, mainly *Azotobacter*, continuously increased from 48 h to 120 h and became one of the dominant groups. Clostridiaceae_1 was slightly enhanced, represented approximately half of the proportion of this family, and gradually decreased until 120 h. Following the addition of *B. cereus* HDYM-02, aerobic or anaerobic pectionolytic bacteria dominated throughout the retting period, resulting in clearly differences in metabolite transitions, particularly galacturonic acid and reducing sugars. In addition to the data on the degumming enzymes and total proteins, the inoculation of *B. cereus* HDYM-02 induced a more thorough degradation of gummy substances. The combination of high-throughput sequencing and the GC-MS technique was effective for monitoring the microbial community and metabolite production, which might offer a better and further understanding of the process and function of retting ecosystems. This study could be helpful to facilitate improvements in the retting process, with shorter times and better fiber qualities.

## Additional Information

**How to cite this article**: Zhao, D. *et al.* Bacterial succession and metabolite changes during flax (*Linum usitatissimum* L.) retting with *Bacillus cereus* HDYM-02. *Sci. Rep.* **6**, 31812; doi: 10.1038/srep31812 (2016).

## Supplementary Material

Supplementary Information

## Figures and Tables

**Table 1:** Summary of the sequencing data sets and statistical analysis of retting solution samples.

Sample	No. of reads	Average read length (bp)	OTUs	ACE	Chao1	Shannon-Weaver
BA-24 h	25034 ± 998	396.28	62 ± 3.51	69 ± 4.16	66 ± 4.00	1.81 ± 0.05
BA-48 h	19507 ± 674	395.93	63 ± 4.04	66 ± 2.64	65 ± 3.21	2.24 ± 0.08
BA-72 h	17604 ± 1557	395.95	73 ± 3.06	82 ± 4.58	77 ± 4.58	2.24 ± 0.05
BA-96 h	20750 ± 1820	396.07	77 ± 4.51	83 ± 4.58	83 ± 4.58	2.47 ± 0.07
BA-120 h	18693 ± 538	396.04	71 ± 3.78	77 ± 3.46	75 ± 4.00	2.04 ± 0.06
sum value of BA	101588 ± 5587^a^		346 ± 12.58^a^	377 ± 18.23^a^	366 ± 20.03^a^	10.8 ± 0.09^a^
CK-24 h	16558 ± 991	395.90	60 ± 3.00	64 ± 3.51	81 ± 3.06	2.20 ± 0.09
CK-48 h	28409 ± 1004	395.89	74 ± 5.67	79 ± 2.08	83 ± 3.51	2.03 ± 0.06
CK-72 h	23826 ± 939	395.95	66 ± 3.06	73 ± 3.06	71 ± 4.58	2.23 ± 0.06
CK-96 h	16584 ± 934	396.00	73 ± 5.03	116 ± 3.51	90 ± 3.60	2.38 ± 0.08
CK-120 h	17747 ± 714	396.03	76 ± 3.06	80 ± 3.00	80 ± 3.60	2.61 ± 0.03
sum value of CK	103124 ± 4583^a^		349 ± 8.08^a^	412 ± 4.36^b^	405 ± 5.29^b^	11.46 ± 0.09^b^

Different letters indicate significant variances between BA and CK samples.

**Table 2:** Loading value of PCA of retting solution samples based on the composition of bacterial communities.

	No. of OTU	Represented family	Represented genus	Loading value
PC1	63 ± 3.01	Clostridiaceae_1	*Clostridium_sensu_stricto_1*	−0.73
	59 ± 2.82	Bacillaceae	*Bacillus*	−0.34
	88 ± 3.86	Pseudomonadaceae	*Azotobacter*	0.52
PC2	38 ± 1.21	Enterobacteriaceae	*Enterobacter*	0.84
	59 ± 1.95	Bacillaceae	*Bacillus*	−0.43

Absolute loading values greater than 0.3 were used to indicate significance among OTUs.
