# Supplementary material for: Bacterial succession and metabolite changes during flax (Linum usitatissimum L.) retting with Bacillus cereus HDYM-02
Source: Sci Rep. 2016 Sep 2;6:31812. doi: 10.1038/srep31812 (PMC5009381; doi:10.1038/srep31812)
Supplement: Supplementary Information [file srep31812-s1.doc]

**Bacterial succession and metabolite changes during flax (*Linum usitatissimum* L.) retting with *Bacillus cereus* HDYM-02**

Dan Zhao^1,2^, Pengfei Liu^1^, Chao Pan^1^, Renpeng Du^1^, Wenxiang Ping^1,2^, Jingping Ge^1,2*^

^1^Laboratory of Microbiology, College of Life Science, Heilongjiang University, Harbin, China

^2^Engineering Research Center of Agricultural Microbiology Technology, Ministry of Education, Harbin, China Heilongjiang University

^*^Corresponding author (gejingping126@126.com)

Supplemental table 1 Abundance of metabolites and multiple comparison based on GC-MS spectra obtained from retting solution samples.

| Chemical Compound | BA 24 h | CK 24 h | BA 48 h | CK 48 h | BA 72 h | CK 72 h | BA 96 h | CK 96 h | BA 120 h | CK 120 h | VIP Value | P Value |
| --- | --- | --- | --- | --- | --- | --- | --- | --- | --- | --- | --- | --- |
| Butanoic acid | 0.0020±0.0001^a^ | 0.0021±0.0003^a^ | 0.0018±0.0000^a^ | 0.0078±0.0002^b^ | 0.0195±0.0005 ^a^ | 0.0197±0.0007^a^ | 0.0308±0.0009^a^ | 0.0192±0.0022^b^ | 0.0181±0.0004^a^ | 0.0301±0.0019^b^ |  |  |
| 4,6-dimethyl-Dodecane | 0.0088±0.0005^a^ | 0.0069±0.0005^b^ | 0.0020±0.0001^a^ | 0.0037±0.0001^b^ | 0.0168±0.0003^a^ | 0.0108±0.0002^b^ | 0.0036±0.0000^a^ | 0.0034±0.0004^a^ | 0.0085±0.0002^a^ | 0.0084±0.0002^a^ | 1.89 | ＜0.05 |
| 2,4-bis(1,1-dimethylethyl)-Phenol | 0.0023±0.0002^a^ | 0.0024±0.0003^a^ | 0.0021±0.0001^a^ | 0.0091±0.0003^b^ | 0.0215±0.0005^a^ | 0.0216±0.0006^a^ | 0.0322±0.0009^a^ | 0.0229±0.0016^b^ | 0.0208±0.0005^a^ | 0.0333±0.0010^a^ | 5.41 |  |
| 2,6-dimethyl-Heptadecane | 0.0012±0.0001^a^ | 0.0012±0.0002^a^ | 0.0011±0.0001^a^ | 0.0040±0.0006^b^ | 0.0122±0.0003^a^ | 0.0121±0.0003^a^ | 0.0173±0.0003^a^ | 0.0132±0.0003^b^ | 0.0121±0.0003^a^ | 0.0185±0.0006^b^ | 3.02 |  |
| Tetradecanoic acid | 0.0138±0.0005^a^ | 0.0118±0.0006^b^ | 0.0071±0.0007^a^ | 0.0121±0.0005^b^ | 0.0280±0.0003^a^ | 0.0172±0.0002^b^ | 0.0051±0.0000^a^ | 0.0058±0.0006^b^ | 0.0146±0.0004^a^ | 0.0133±0.0001^b^ | 2.52 | ＜0.05 |
| Phthalic acid | 0.0055±0.0007^a^ | 0.0071±0.0016^b^ | 0.0049±0.0003^a^ | 0.0050±0.0008^a^ | 0.0277±0.0003^a^ | 0.0172±0.0007^b^ | 0.0055±0.0001^a^ | 0.0052±0.0002^b^ | 0.0132±0.0006^a^ | 0.0129±0.0003^b^ | 1.23 | ＜0.05 |
| n-Hexadecanoic acid | 0.0017±0.0002^a^ | 0.0023±0.0004^b^ | 0.0030±0.0002^a^ | 0.0014±0.0001^b^ | 0.0087±0.0003^a^ | 0.0069±0.0002^b^ | 0.0053±0.0001^a^ | 0.0034±0.0001^b^ | 0.0028±0.0002^a^ | 0.0049±0.0002^b^ | 2.01 | ＜0.05 |
| 1-(9-anthracenyl)-Ethanone | 0.0076±0.0004^a^ | 0.0107±0.0005^b^ | 0.0222±0.0004^a^ | 0.0138±0.0002^b^ | 0.0064±0.0003^a^ | 0.0105±0.0003^b^ | 0.0107±0.0004^a^ | 0.0128±0.0001^b^ | 0.0083±0.0003^a^ | 0.0122±0.0003^b^ | 1.27 | ＜0.05 |
| 4-(2-propenyl)-Phenol | 0.0005±0.0000^a^ | 0.0007±0.0001^b^ | 0.0009±0.0001^a^ | 0.0005±0.0000^b^ | 0.0044±0.0002^a^ | 0.0036±0.0001^b^ | 0.0025±0.0000 ^a^ | 0.0018±0.0001^b^ | 0.0014±0.0001^a^ | 0.0024±0.0001^b^ | 1.34 | ＜0.01 |
| 6-Octadecenoic acid | 0.0050±0.0001^a^ | 0.0077±0.0002^b^ | 0.0098±0.0001^a^ | 0.0100±0.0000^a^ | 0.0037±0.0001^a^ | 0.0067±0.0001^b^ | 0.0045±0.0001^a^ | 0.0074±0.0001^b^ | 0.0051±0.0001^a^ | 0.0053±0.0001^a^ | 2.61 | ＜0.05 |
| Octadecanoic acid | 0.0090±0.0003^a^ | 0.0080±0.0006^b^ | 0.0045±0.0003^a^ | 0.0080±0.0003^b^ | 0.0180±0.0002^a^ | 0.0107±0.0002^b^ | 0.0029±0.0000^a^ | 0.0038±0.0002^b^ | 0.0100±0.0004^a^ | 0.0082±0.0002^b^ | 2.03 | ＜0.01 |
| carboxylic acid ethyl ester | 0.0053±0.0006^a^ | 0.0044±0.0005^b^ | 0.0004±0.0001^a^ | 0.0007±0.0001^b^ | 0.0002±0.0000^a^ | 0.0006±0.0000^b^ | 0.0001±0.0000^a^ | 0.0005±0.0000^b^ | 0.0008±0.0000^a^ | 0.0001±0.0000^b^ | 1.00 | ＜0.05 |
| Methyl dehydroabietate | 0.0141±0.0008^a^ | 0.0106±0.001^b^ | 0.0015±0.0001^a^ | 0.0015±0.0003^a^ | 0.0009±0.0001^a^ | 0.0025±0.0001^b^ | 0.0006±0.0000^a^ | 0.0012±0.0001^b^ | 0.0029±0.0000^a^ | 0.0007±0.0000^b^ | 2.73 | ＜0.05 |
| Tetracosane | 0.0033±0.0002^a^ | 0.0048±0.0002^b^ | 0.0095±0.0002^a^ | 0.0064±0.0001^b^ | 0.0023±0.0001^a^ | 0.0041±0.0001^b^ | 0.0037±0.0002^a^ | 0.0056±0.0004^b^ | 0.0037±0.0002^a^ | 0.0045±0.0002^b^ | 2.50 | ＜0.01 |
| 9-methyl-Nonadecane | 0.0109±0.0006^a^ | 0.0024±0.0002^b^ | 0.0008±0.0000^a^ | 0.0013±0.0000^b^ | 0.0003±0.0000^a^ | 0.0006±0.0000^b^ | 0.0002±0.0000^a^ | 0.0010±0.0001^b^ | 0.0015±0.0001^a^ | 0.0004±0.0000^b^ | 2.41 | ＜0.01 |
| 2,6-dimethyl-1,4-dinitroso-Piperazine | 0.0062±0.0003^a^ | 0.0089±0.0003^b^ | 0.0177±0.0001^a^ | 0.0088±0.0001^b^ | 0.0053±0.0002^a^ | 0.0050±0.0001^a^ | 0.0071±0.0003^a^ | 0.0074±0.0007^a^ | 0.0064±0.0002^a^ | 0.0030±0.0002^b^ | 1.85 | ＜0.05 |
| Phthalic acid, di(2-propylpentyl)ester | 0.0058±0.0002^a^ | 0.0026±0.0001^b^ | 0.0004±0.0001^a^ | 0.0030±0.0000^b^ | 0.0002±0.0000^a^ | 0.0010±0.0000^b^ | 0.0001±0.0000^a^ | 0.0011±0.0001^b^ | 0.0009±0.0000^a^ | 0.0002±0.0000^b^ | 1.93 | ＜0.01 |
| 2,6-Di-t-butyl-4-dimethylaminophenol | 0.0075±0.0005^a^ | 0.0015±0.0002^b^ | 0.0005±0.0001^a^ | 0.0009±0.0000^b^ | 0.0002±0.0000^a^ | 0.0004±0.0000^b^ | 0.0001±0.0000^a^ | 0.0007±0.0001^b^ | 0.0009±0.0001^a^ | 0.0002±0.0000^b^ | 1.92 | ＜0.05 |
| Squalene | 0.0107±0.0005^a^ | 0.0062±0.0002^b^ | 0.0001±0.0001^a^ | 0.0023±0.0000^b^ | 0.0005±0.0000^a^ | 0.0013±0.0000^b^ | 0.0000±0.0000^a^ | 0.0014±0.0002^b^ | 0.0031±0.0000^a^ | 0.0001±0.0000^b^ | 3.06 | ＜0.05 |

Different letters indicate significant variances between BA and CK samples at each time point.
